# Science serving justice: opportunities for enhancing integrity in forensic science in Africa

**DOI:** 10.1080/20961790.2021.1989794

**Published:** 2021-11-24

**Authors:** Antonel Olckers, Zoë Hammatt

**Affiliations:** a DNAbiotec (Pty) Ltd, Pretoria, South Africa; b University of Hawaii and Z Consulting, Honolulu, Hawaii, USA

**Keywords:** forensic science, forensic science laboratory, research integrity, Africa; bias, oversight, networks, mentoring

*If the law has made you a witness, remain a man [practitioner* ^1^ *] of science. You have no victim to avenge, no guilty or innocent person to convict or save—you must bear testimony within the limits of science.* ^2^

Forensic science is a unique discipline of applied science. At its core it is a science, yet finds its use in the application to cases that often go to a court of law. Practicing at the intersection of science and law poses distinct challenges, but also holds incredible potential in its ability to serve as a vehicle for integrity. The practice of forensic science in Africa is at a tipping point. Unchartered waters lie ahead for the continent and its forensic scientists. Often working in inadequately resourced environments, obstacles may seem insurmountable. As African forensic scientists, we can overcome these obstacles as we accept that we are connected and united in our goal to allow science to serve justice.

With this article, we seek to shed light on building a strong forensic science research culture and practicing with integrity in Africa, whether in court, forensic science laboratories (FSLs), independent practice, or as mentors and international collaborators. While a few key aspects are touched upon in the African context, there are many other challenges and opportunities in our daily work that remain to be explored.

## Forensic science defined

The 2021 International Association of Forensic Sciences’ (IAFS) Sydney Declaration [1] defines forensic science as follows:

*Forensic science is a case-based (or multi case-based) research-oriented endeavour using the principles of science to study and understand traces—the remnants of past activities (such as an individual’s presence and actions)—through their detection, recognition, examination and interpretation to understand anomalous events of public interest (e.g., crimes, litigations, security incidents).*

The phrase describing the field as a “*case-based research-oriented endeavour*” emphasises that research is an integral component of forensic science. In addition, the seven principles in the IAFS Sydney Declaration highlights the need for the diverse set of skills and reasoning models that are essential to our practice (see below).

Principle 1Activity and presence produce traces that are fundamental vectors of information.Principle 2Scene investigation is a scientific and diagnostic endeavour requiring scientific expertise.Principle 3Forensic science is case-based and reliant on scientific knowledge, investigative methodology and logical reasoning.Principle 4Forensic science is an assessment of findings in context due to time asymmetry.Principle 5Forensic science deals with a continuum of uncertainties.Principle 6Forensic science has multi-dimensional purposes and contributions.Principle 7Forensic science findings acquire meaning in context.

By following these principles, we can ensure that science remains at the core of our practice and that our practice is research based.

## The scientific method and cognitive bias

The scientific method has at its core the testing of a scientific hypothesis and is understood and followed diligently by ethical forensic scientists. Voit [2] argues that there are three dimensions to the scientific method, however, it is the traditional hypothesis-based deductive reasoning model that is currently embraced in forensic science. Inductive reasoning is used as envisaged by Kind [3] where appropriate in a case, or as a blended approach between inductive and deductive reasoning models when appropriate. When the scientific method is followed, it is logical that a conclusion is reached at the end, only after formulating and testing a hypothesis.

In the seven principles, the IAFS Sydney Declaration of May 2021 [1] encapsulates the scientific method in a practical manner as it applies to forensic science.

Underlying these principles, the scientific method dictates that all evidence be considered—no doubt an attempt to manage factors such as the cognitive bias inherent in humans. Ignoring the scientific method and cognitive bias in forensic science is highly detrimental as it destroys the integrity of the results generated. Camilleri et al. [4] recognised that cognitive bias is a threat in forensic science practice and developed and implemented a risk-based cognitive bias management tool to address such bias with, for instance, the elimination of non-relevant case contextual information. In the DNA evidence field, sequential unmasking is one of the commonly used tools for minimising the effects of cognitive bias [5,6].

As Dror and Kukucka [7] recently pointed out, the order in which information is presented affects decision making and conclusions drawn in forensic science. They formulated the Linear Sequential Unmasking-*Expanded* (LSU-*E*) model, which reduces noise and improves decision making. The three criteria for this model are: biasing power, objectivity, and relevance. In addition, the LSU-*E* model is not limited to comparative analyses and can thus be used in any field of forensic science.

To serve justice, it is our responsibility in Africa and elsewhere to diligently follow the scientific method while managing bias, relevance, and objectivity to the greatest extent possible.

## Good stewards of science: the Singapore Statement

As forensic scientists we are entrusted to be good stewards of our field. This concept was articulated for all disciplines by the World Conferences on Research Integrity Singapore Statement on Research Integrity (WCRI Singapore Statement) [8]. Complementing the IAFS Sydney Declaration, its four main principles provide an excellent guide for the practice of forensic science and are summarised as follows:

***Honesty*** *in all aspects of research*

***Accountability*** *in the conduct of research*

***Professional courtesy and fairness*** *in working with others*

***Good stewardship*** *of research on behalf of others*

Unfortunately, the lack of accountability for forensic scientists is pervasive in the South African system. One reason for this is that there are too few independent and credentialed forensic scientists who can verify the work of others and assist the courts. This problem persists throughout Africa.

Whether addressing the principles of the Singapore Statement such as accountability or the subtle nuances that emerge related to objectivity and independence, following professional codes and ethical tenets is perhaps one of our primary avenues for serving justice responsibly.

## Professional and ethical practice

Professionalisation of forensic science practice would help ensure that practitioners follow a code of conduct, are nationally regulated, and understand behaviours that could erode their integrity. In South Africa, the South African Academy of Forensic Sciences (SAAFS), was established in 2018 as our forensic science professional body and for the first time made adherence to a professional Code of Conduct compulsory for its members [9].^3^ Professionalisation [10] is the first step toward ensuring accountability on behalf of the practicing forensic scientists in the country. To this end, SAAFS is the only body that represents forensic scientists from all fields and sectors in South Africa. One of its primary goals is to encourage the development and maintenance of forensic science and ethical standards, all of which are intricately linked to principles of integrity and trustworthiness.

Unethical practice destroys the validity and value of any resulting scientific evidence. We and others have pointed out that in the DNA evidence field alone, the limitations range from contamination, secondary transfer, cognitive bias, statistical manipulation, misinterpretation, analysis errors, testimony beyond the limits of science, to other aspects of unethical practice. Professionals should be aware of the implications of these complex issues. It is our ethical responsibility as forensic scientists to indicate the potential of specific tools in our field, such as DNA evidence, but also to mention their limitations. To this end, problems such as error rates and uncertainty of measurement statements must be presented.

## Regulation, certification, accreditation, and oversight

Forensic scientists and their laboratories must work at an acceptable standard to foster confidence in the work performed. Our work should be on par with best practice globally. To this end, the regulation of the forensic science profession is vital.

Unfortunately, some individuals call themselves “forensic scientists” and practice as such when they are not qualified in the field. There are some certification bodies for forensic scientists in Africa, but not nearly enough. In some countries, bodies exist where forensic scientists can be certified to work in a specific field. This is equally important, as the certification body is often specific to a sub-specialty field in forensic science and has prescribed codes to which members of the sub-speciality must adhere.

Professional oversight ensures that practicing forensic scientists are appropriately trained and qualified and remain abreast of advances in their field through continued professional development. For those countries where this is not yet a reality, it is imperative to affiliate with professional bodies that require adherence to an appropriate code of conduct. Regulation of the forensic science profession, however, should still be pursued as the ultimate goal. This not only builds trust in the justice system among members of society, but also affords professional recognition to the practicing forensic scientist in his/her/their own jurisdiction and beyond.

Throughout Africa, forensic science laboratories are being set up or expanded operationally every year. Some of the national laboratories on the continent have been operational for decades, with others only starting or still in the conceptualisation phase. Standards for the responsible practice of forensic science hold true for a forensic laboratory regardless of the stage of operation or conceptualisation.

Accreditation of FSLs is critical to ensure that the minimum standards prescribed in the field are followed (i.e. accreditation according to ISO 17025, which assures that calibration and testing laboratories are delivering good services and consistent data are being produced). Simply stating that a laboratory “complies with the standard” is not enough if no external evaluation has been performed to verify such compliance. In South Africa, the South African National Accreditation System (SANAS) [11] is legally mandated to accredit laboratories in the field of forensic science. SANAS also accredits laboratories all over Africa, and many other countries use SANAS for this purpose. In Southern Africa, some countries use the Southern African Development Community Accreditation Services (SADCAS) [12] for accreditation. Regardless of which accrediting body is used, the FSLs must seek accreditation as early as possible to ensure not only that the lab has been independently reviewed for adherence to international standards, but also that its results are comparable to results generated in other jurisdictions.

Crimes do not respect borders. As such, it is often necessary to compare forensic science results with other FSLs in Africa or globally, necessitating formal recognition according to a common and appropriate standard. Accreditation is also the best assurance to a court of law as well as victims and their families, defendants, and the public at large, that a particular laboratory practices according to the minimum standard in the field. An unaccredited national laboratory can produce negative consequences on several levels, contrasting directly with the ethos of Science Serving Justice. Detrimental effects of the lack of accreditation can be further compounded by the lack of regulation in the forensic science profession [13].

These challenges must be handled transparently and decisively. Other jurisdictions have been able to achieve this [14,15], which in the UK, for example, led to the founding of the Forensic Science Regulator [16], an independent body responsible for standards generation, compliance verification, advice and guidance. The victims of crime in Africa deserve no less.

The WCRI Singapore Statement outlines 14 principles that should be incorporated in the standard operating procedures of FSLs. Doing so allows for corrective and even punitive actions when the procedure is not followed, but more importantly informs the entire workforce in the FSLs about the appropriate standards to apply in their practice. Principle 13 states that a workplace should “*encourage integrity through education, clear policies, and reasonable standards for advancement, while fostering work environments that support research integrity*” [8]. This critical aspect should be clearly articulated in institutional policies of forensic science laboratories.

In addition, we have long been advocating for an independent oversight body in forensic science in South Africa [17]. The investigating arm and the national forensic science laboratory are part of the same entity, the South African Police Service, with the prosecuting arm residing under the Department of Justice. A small number of forensic testing laboratories reside under the Department of Health—highlighting the aspect of fragmentation of forensic services, another important issue to address, as outlined by Roux et al. [18]. Africa is not unique in this regard, as most national forensic science laboratories are linked, or reside under, national police departments. To eliminate any real or perceived conflict of interest or influence, a forensic science laboratory should be a wholly independent entity. Where this is not possible, potential conflicts can be managed if stringent disclosure and management systems are in place.

All of the above aspects work in harmony to ensure that robust and independent forensic science evidence can be generated to serve justice. Missing one of these key mechanisms in a national forensic science ecosystem will undermine the value that forensic science may offer to the justice system.

## Contrasting roles for science, law and the media in court cases

The roles of the expert forensic science witness, the legal professionals advocating for either the prosecution or defence, and the presiding officer in the court, are distinctly different, as indicated in Table 1. Although each legal professional is duty bound to act with integrity in the pursuit of justice, each role assumes a primary responsibility and emphasis. The presiding officer serves as a fact finder who applies the law to deliver a just verdict. While the other legal professionals also seek to administer justice as officers of the court, they advocate for their case: prosecutors for the State; and the defence for their client(s). In South Africa the presiding officer can be a magistrate or judge, depending on the type of court, and fulfils the role the jury would play in other jurisdictions. This presiding officer hears the case and delivers the judgement and sentencing.

**Table 1.** Roles of different professionals in a court of law.

Professional	Responsibility	Role
Forensic scientist/expert witness	Science	Neutrally assist the court
Legal: prosecutor	State	Prosecute the case
Legal: defence advocate	Accused	Advocate for client
Legal: presiding officer (judge or magistrate)	Justice	Deliver a verdict based on law
Media	Public interest	Report on the case

Although there are nuances inherent to each of the above roles, the forensic scientist’s fundamental obligation is to assist the court. The issue of guilt or innocence is immaterial in this context. When we contract with legal professionals, our mantra is clear: “*what we have to say can help or hurt your case/client, as long as it is understood that these two outcomes are equal to us*”. As a forensic scientist, we do not testify “on behalf of” any party involved, whether prosecution or defence. We are neutral providers of scientific fact and are there to assist the court by clarifying the often technically complex aspects involved in the scientific data submitted as evidence. It is thus essential to ensure that our actions and testimony as expert witnesses do not contribute to potential cognitive biases or infringe upon the integrity of the evidence presented. Particularly in the adversarial court environment, we must maintain our resolve in the face of strategies used by legal professionals, many of whom seek to discredit the witness if there are problems with the scientific evidence presented in their case.

It is dangerous for forensic scientists to stray outside the bounds of their role in court by advocating the case of the prosecution or defence. This can occur because of institutional or individual cognitive bias or other undue pressure placed on the scientist. In some jurisdictions and forensic science laboratory environments, this pressure is exacerbated when inappropriate performance metrics are used, such as the conviction rate, to incentivise or reward forensic scientists. Regarding this perverse incentive in the context of prosecutors in South Africa, the Chief Justice of South Africa [19] emphasised that:

*… reliance or the requirement for prosecutors to rely on the conviction rate as a performance yardstick must be corrected. They don’t convict. Judicial Officers do. How then can it ever be appropriate to measure their performance on the basis of what they don’t do? Theirs is to present cases, and even support an acquittal where the interests of justice would be served by doing so. Not to pursue a conviction at all costs.*

This message is equally relevant to forensic scientists, who are also not judicial officers. Inappropriate performance metrics, incentives and rewards should be discouraged and eliminated as they run counter to the ethical and integral practice of forensic science. The WCRI Hong Kong Principles, formulated in 2019 to help minimise perverse incentives, offer an excellent guide for recognising and rewarding researchers for behaviours that strengthen integrity, such as complete reporting [20].

The media can also play a role in serving justice. This is even more critical as the media increasingly becomes involved in court cases beyond merely reporting in the printed press [21]. In South Africa there have been several High Court cases where the Court allowed the media to livestream the entire court proceeding. The live streaming and social media posting often make the expert witness the focus of the news. If the media is afforded the privilege of being present in court, emphasis should be placed on reporting on the case itself rather than on trivial matters. Interacting with the press can be difficult, especially during an adversarial court proceeding. In responding to questions from the media, the expert witness can offer educational responses about forensic science without divulging specifics of the case. This can help prevent sensationalism as well as contribute to expanded public knowledge.

## Expert witness integrity

Whether testifying in court or communicating in any professional setting, part of our integrity emerges from being honest, which is also highlighted in the WCRI Singapore Statement. We are human—this means we make mistakes. When this happens, regardless of the forum, we must acknowledge our mistake, correct it, and move on. Not only does this establish our credibility, it reinforces trust in our word and in the process.

Another element in the process is payment to expert witnesses, which is sometimes questioned. All professionals in court are paid, and payment to forensic science experts is acceptable. Such payment should of course be reasonable and determined by appropriate standards. As part of their professional integrity, experts should also make clear that their testimony will reflect an unbiased analysis of the data, not a particular outcome.

The forensic expert witness is often faced with challenging questions in court. The defence advocate and prosecutor always follow a strategy. Expert witnesses should try to understand this strategy as early as possible under cross-examination. Describing the scientific evidence early can sometimes help circumvent cross-examination tactics often used by lawyers. The expert witness should have a clear focus in mind prior to stepping into the witness box; namely, to assist the court by telling the truth and serving as an objective and responsible steward of science.

Another way to serve justice as an expert witness is to use pre-trial conferences. They are not commonly used in Africa, despite being particularly productive for criminal cases by enabling forensic science experts to resolve issues on which they agree and save only disputed issues for court. Although we may request such pre-trial conferences, they are often denied. In South Africa, legal professionals cite two reasons for resisting this practice: that the defence has no pre-trail disclosure obligation, and the preference for retaining the element of surprise in court. Each of these reasons can be frustrating for the expert witness called by the defence in a trial, but trial strategy is the purview of the legal professionals. While advising policy makers to update legislative frameworks and mandate this practice, we can advocate for a pre-trial conference in every case, which would make our justice systems more efficient and expedient.

## A call to action for integrity in forensic science laboratories in Africa

There are only a few well-established FSLs in Africa. Many countries are just now bringing their FSLs into operation. Even established FSLs in Africa are fraught with problems, ranging from cognitive bias, lack of competence, and corruption, which must be confronted, regardless of how uncomfortable it may be. Countries setting up their FSLs should be cognisant of issues faced by other laboratories across the continent and try to avoid mistakes through careful planning. Sharing experiences openly with other forensic scientists in Africa can shed light on lessons learnt. Some of our problems can be addressed with technology (e.g. reducing backlogs or adding new technologies for more efficient processing), as well as training and education.

Other concerns, such as toxic work environments and unethical practice, are equally complex. These can be addressed through transparency, solidarity, and monitoring of stated commitments to ethical practice and integrity, with meaningful measurement of progress, and political will at all levels. These concerns are clearly not unique to Africa. Nevertheless, they give rise to an opportunity to craft an African approach to designing innovative solutions as we join forces with others who share a common vision of integrity in the forensic sciences in Africa.

As noted, forensic science is a research-based endeavour. If capacity is lacking, a forensic science laboratory need not necessarily run its own research facility and can instead forge links with nearby universities and institutes. Rwanda provides a stellar example with their “Knowledge transfer for forensic science development” project established in 2012 [22]. Among others, the project involves the Rwanda Forensic Laboratory and the University of Rwanda, and supports knowledge exchange between the lab, the university, the Institute of Legal Medicine, the National Police College in Rwanda, and international collaborators, as well as interaction with the public prosecutor’s office. The project was established to fulfil the “need for further training of forensic scientists” [22]. Complementing technical skills in forensic science, discussions around mental health are offered through the Center for Mental Health at the University of Rwanda. This blend of hard technical and soft skill enhancement offers a holistic approach to fostering healthy work environments in FSLs while producing scientists who are well rounded and capable of making ethical decisions.

## Embracing technology and keeping up to date

Practicing forensic science with integrity implores the scientist to remain abreast of developments in one’s field of practice and adapt methods to stay on par with current international best practice. In this regard, a system of compulsory continued professional development is invaluable. Clinging to out-dated methods and practices results in a disservice to society. Change is a constant element. Its presence forces us to continually evolve as scientists, understanding new technologies and adapting to paradigm shifts. As such, we should constantly reform our profession, adopting global best practices and unlocking the full potential of forensic science throughout Africa.

Technologies such as machine learning pose incredible opportunities for forensic scientists of Africa and elsewhere. All methods of course have limitations, and algorithms must be developed, evaluated, and monitored by those with relevant expertise to minimise potential for bias. Nevertheless, there are innovative hubs of excellence in software development in Africa. We should reach out and share resources, exchanging ideas and knowledge with others as they develop the software and other tools that we use.

Another issue worth noting around technological solutions in Africa is that once they are instituted to eliminate the backlogs that plague the forensic science sector, for example in DNA testing, these backlogs will translate to the justice system. Engaging legal professionals as we design forensic science backlog solutions is important, as courts may be flooded with cases once the testing backlogs are cleared. It is our duty to contribute collaboratively to solutions for managing future delays in our justice systems.

## Enhancing integrity through networks

Forensic scientists do not practice in isolation. Like all scientists, we form part of and function within an ecosystem. We should network with others within this ecosystem and ensure that our work can go to court and thereby enable science to serve justice. This entails networking across multiple disciplines. We should ensure that current legal practitioners are aware of the advantages as well as the limitations of our fields. Van der Merwe et al. [23] deployed a programme of training legal professionals to serve this goal. We encourage this approach in other jurisdictions as legal practitioners are empowered to deal with forensic science evidence in court.

As noted above, the South African Academy of Forensic Science (SAAFS) was founded in 2018 [24]. Among its goals are to “*encourage the development and maintenance of forensic scientific and ethical standards, to encourage research in the forensic sciences, to encourage co-operation and to deal with all such matters as may affect their common interests in respect of forensic sciences*” [9]. Its spirit of co-operation has been demonstrated by its well-attended series of webinars where forensic scientists from all over Africa can join a neutral discussion platform each month to meet professional colleagues and discuss relevant issues. These webinars are also attended by an international audience, which enriches the discussion and reminds us that most of the challenges we face in forensic science are not unique to Africa.

It is helpful to have independent bodies conduct consensus studies that consult with forensic scientists to define what is lacking in our field, and reveal how we can network across traditional disciplines to enhance our practice. One such study by the Academy of Science of South Africa (ASSAf) [25] found that independent oversight, accountability and transparency, public awareness about DNA testing, incorporation of DNA evidence in law curricula, legislative updates, and sanctions for non-compliance, need to be addressed in the forensic science context in South Africa. This study focussed on the ethical, legal and social implications of human genetics and genomics in South Africa, and thus only addressed a few issues in terms of DNA evidence. If each jurisdiction in Africa were to conduct similar consensus studies for the forensic sciences, this could generate recommendations and outline next steps for improvements.

Ideally, forensic science networks in Africa should also include discussion with legal professionals and public regulators, as is common in the rest of the world. This can accelerate the pace of legislative reform, ensuring that developments in our field are translated into action in our justice systems. In this way, those of us practicing at the intersection of science and law can align accordingly with the knowledge and insight science can offer to the court and justice system.

Other African networks such as the African Academy of Sciences (AAS) [26] and the African Research Integrity Network (ARIN) [27] can offer new perspectives as we build a positive research culture together in Africa. Ultimately those countries with established forensic science professional societies should consider uniting under a body that we all envisage as an African Academy for Forensic Sciences.

## The next generation of forensic scientists in Africa

When looking at Africa, one is struck by the high level of unemployed high school graduates across the continent. Although tertiary education partially addresses this problem, a mismatch between skills and jobs performed was reported for employed graduates in South Africa [28]. At least in South Africa, our graduates are not “market ready”. Roux et al. [18] indicated that the same is true for forensic science graduates globally. In establishing new forensic science programmes and curricula, we should endeavour to educate forensic scientists who are ready to embark on successful careers in their chosen profession. This means ensuring that they are trained in rigorous scientific methods as well as ethics and responsible research practice. Involvement in research partnerships between FSLs and universities can also help prepare graduates for their careers by exposing them to practical challenges in the research setting.

We must equip students with basic skills such as logical thinking and deductive reasoning, training them to understand models of thought and ethical deliberation. To prepare the learner for a science, technology, engineering, mathematics and innovation (STEMI) field, including forensic science, we must adjust our education systems to teach upcoming forensic scientists in Africa the attributes they need to excel and contribute to the broader society.

Forensic science is not yet widely established as an independent curriculum with graduate degrees at all levels. Often those with degrees in science migrate to forensic sciences after graduation, without the appropriate background in legal proceedings and the law. In 2012, Roux et al. [18] highlighted that “*skill deficits about basic forensic science theory and professional attitudes*” are problematic for employers. Roux and colleagues noted that “*distinctive tertiary education in forensic science*” has been called for over the past 100 years [18]. This call to action should be heeded.

For young professionals, especially recent graduates, it is not easy to enter our profession. They need experience to become registered and join professional bodies as forensic practitioners. Mentoring is key to affording young professionals the chance to acquire skills and experience. In addition to offering guidance throughout the process of working on a case, mentors can instil a culture of lifelong learning and integrity in practice.

It is also time for us to reach across national borders within Africa to mentor and prepare our young graduates. Their talent should be nurtured and career paths with ample opportunity for growth should be the norm. Given the workload of high-throughput FSLs, this can be difficult. The question is often asked, can we afford to take forensic analysts away from their tasks to attend yet another training event? In our view the more relevant question is, can we afford *not* to offer training opportunities for our young forensic scientists?

## Conclusion

In Africa, we need to raise awareness of the “research-based” component inherent to forensic science practice. Roux et al. [18] described the absence of a research culture in forensic science as a “*major flaw in the system*”. By serving as good stewards of science, remaining accountable to ourselves and to each other, diligently following the scientific method and reducing cognitive bias wherever possible, we are taking our first steps toward creating a thriving research culture in forensic science. Professionalising our practice and staying true to our role as expert witnesses is yet another dimension. Ensuring that our forensic science laboratories are operating at appropriate standards and reaching out across the continent and globally through networks will take us even further. Perhaps most critically, we are responsible for sharing our knowledge by mentoring the next generation of forensic scientists. In so doing, we can uplift the field by aligning with the principles of integrity and allowing science to serve justice.


Antonel Olckers 
*DNAbiotec (Pty) Ltd, Pretoria, South Africa *


Zoë Hammatt 
*University of Hawaii and Z Consulting, Honolulu, Hawaii, USA*




## Notes

1 Practitioner in this context indicates he/she/they.

2 Quote by Dr. Paul CH Brouardel, 19th Century French medico-legalist.

3 Antonel Olckers is a Founding Director, Full Member (Biology/DNA) and the Chair (May 2018 to present) of SAAFS.
